# An overview of machine learning and deep learning techniques for predicting epileptic seizures

**DOI:** 10.1515/jib-2023-0002

**Published:** 2023-12-15

**Authors:** Marco Zurdo-Tabernero, Ángel Canal-Alonso, Fernando de la Prieta, Sara Rodríguez, Javier Prieto, Juan Manuel Corchado

**Affiliations:** BISITE Research Group, University of Salamanca, Salamanca, Spain

**Keywords:** seizure prediction, machine learning, epilepsy, electroencephalogram

## Abstract

Epilepsy is a neurological disorder (the third most common, following stroke and migraines). A key aspect of its diagnosis is the presence of seizures that occur without a known cause and the potential for new seizures to occur. Machine learning has shown potential as a cost-effective alternative for rapid diagnosis. In this study, we review the current state of machine learning in the detection and prediction of epileptic seizures. The objective of this study is to portray the existing machine learning methods for seizure prediction. Internet bibliographical searches were conducted to identify relevant literature on the topic. Through cross-referencing from key articles, additional references were obtained to provide a comprehensive overview of the techniques. As the aim of this paper aims is not a pure bibliographical review of the subject, the publications here cited have been selected among many others based on their number of citations. To implement accurate diagnostic and treatment tools, it is necessary to achieve a balance between prediction time, sensitivity, and specificity. This balance can be achieved using deep learning algorithms. The best performance and results are often achieved by combining multiple techniques and features, but this approach can also increase computational requirements.

## 1 Introduction

Epilepsy is a neurological disorder that affects over 39 million people in the United States [1], making it the third most common disorder after stroke and migraines [2]. This chronic condition is known to impact the quality of life significantly, making it a focus of ongoing research and development of new therapies. According to the International League Against Epilepsy, an epileptic seizure is a “transient occurrence of signs and/or symptoms due to abnormal excessive or synchronous neuronal activity in the brain” [3].

**Table 1:** List of the features that can be extracted for seizure prediction and detection.

Time domain	Kurtosis
	Mean
	Skewness
	Variance
	Phase Correlation
	Common Spatial Pattern
Nonlinear features	Correlation dimension
	Largest Lyapunov Exponent
	Fractality Dimension
	Lempel-Zic Complexity
	Approximate Entropy
	Spectral Entropy
	Wavelet Entropy
	Hjort Parameters Analysis
	Wavelet Energy
Frequency Domain Features	Short Time Fourier Transform
	Fractional Fourier Transform
	Spectral Power Analysis
Time-frequency domain features	Discrete Wavelet Transform
	Continuous Wavelet Transform
	Higher-Order Spectra
	Variational Model Decomposition

**Table 2:** Feature extraction application.

Time domain	Parvez and Paul [9]
Nonlinear features	Aarabi et al. [12]
	Shafique et al. [14]
	D’Alessandro et al. [16]
	Zhang et al. [19]
	Direito et al. [21]
	Gadhoumi et al. [23]
Frequency Domain Features	Truong et al. [25]
	Bandarabnadi et al. [28]
Time-frequency domain features	Ouyang et al. [35]
	Acharya et al. [32]
	Kumat and Rao [34]

**Table 3:** Feature selection methods.

Principal Component Analysis (PCA)	Williamson et al. [36]
Partial Least Squares (PLS)	Varatharajah et al. [45]
Minimum Redundancy Maximum relevance (MRMr)	Bandarabadi et al. [39]
Gaussian Mixture Models	Zandi et al. [41]
Deep Learning (Automatic Feature Selection)	Wei et al. [43]
	Yan et al. [44]
	Tsiouris et al. [46]

**Table 4:** Seizure prediction methods.

Author	Features	Method	Pred. time	Results
***Animal models***				
Ouyang et al. [35]	Wavelet transform	Wavelet-based nonlinear similarity index	2.14 min	Sensitivity > 90 % Specificity = 6.25 %
Varatharajah et al. [45]	Power in band (PIB) features, time domain correlations (TMCO), and spectral coherence (SPCO) between different pairs of channels	Support Vector Machine, Artificial Neural Networks, and Random Forests Classifier	1 min	Specificity = 70 % Specificity = 75 %
Rajdev et al. [49]	Filtered signal	Adaptive Wiener algorithm	6.7 s	Sensitivity = 94 % False positive rate (FPR) = 0.009 min^−1^
***Human subjects***				
Iasemidis et al. [50]	Short-term maximum Lyapunov exponent	Spatial dynamical entrainment of short-term maximum Lyapunov exponent	89 min	Sensitivity = 91.3 % FPR = 8.27 h^−1^
Aarabi et al. [12]	Correlation dimension, correlation entropy, noise level, Lempel-Ziv complexity, largest Lyapunov exponent, and nonlinear inter-dependence	Patient-specific rule-based algorithm	50 min	Sensitivity >90 % FPT <0.15 h^−1^
Zhang et al. [19]	Higuchi Fractal Dimension	Bayesian linear discriminant analysis as a classifier	39.39 min	Sensitivity = 89.33 % FPR = 0.20 h^−1^
Tsiouris et al. [46]	Time domain, Frequency domain, Brain connectivity, and Graph-theoretic features	LSTM	120 min	Sensitivity > 99 % and FPR <0.06 h^−1^
Wei et al. [43]	CNN automatic feature extraction	Long-Term Convolutional Neural Network	21 min	Sensitivity = 93.40 % Specificity = 86.13 %
Yan et al. [44]	STFT	Transformers	30 min	Sensitivity = 96.01 % FPR = 0.047 h^−1^

**Figure 1:**
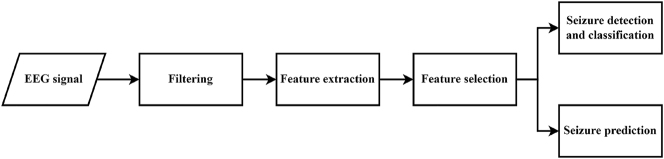
The standard process for classifying or predicting epileptic seizures.

### 1.1 Issues

Electroencephalography (EEG) is the most commonly used method for diagnosing epilepsy. This technique involves recording the brain’s electrical activity through the measurement of voltage changes caused by the ionic currents of brain cells [4]. Electrodes placed on the scalp (sEEG) or inside the cranium (iEEG) are used to record the voltage changes produced by the brain’s electrical activity. These voltage changes result from the simultaneous activity of millions of neurons, which generate a voltage wave that the electrodes can detect.

The events observed in the EEG can be grouped into three categories [5]: ictal events, which occur during a seizure; preictal events, which occur before a seizure; and interictal events, which are all other events that are not part of the ictal or preictal phase. The duration of the preictal phase can vary widely, from a few minutes to several hours.

In animal and *in vitro* models, microelectrodes are sometimes used instead of EEG to perform electrophysiology recordings, due to the limitations of EEG in these contexts.

### 1.2 Objectives

This study aims to give an overview of the existing machine-learning techniques for predicting epileptic seizures based on features extracted from EEG signals. As it is not intended to portray a pure bibliographical review of the subject, the publications here cited have been selected among many others based on their relevance and number of citations.

The present document goes through the usual pipeline for seizure prediction (Figure 1). First of all, the processing of the EEG signal will be discussed, including artifact-removal techniques. Later, we will discuss the most relevant features that can be extracted from the preprocessed signal, and how we can manage to select those that contain the optimal amount of information needed to generate a predictive model. Finally, the most prevalent methods for epileptic seizure prediction will be exposed, separating studies using animal and human EEG.

### 1.3 Questions and hypothesis

The use of machine learning (ML) and deep learning (DL) methods for epileptic seizure detection and prediction has gained significant attention in recent years. However, several questions and hypotheses remain unanswered. In this paper, we aim to address the following questions and hypotheses:

– **Which features are most relevant for epileptic seizure detection and prediction?** Various features have been proposed for ML and DL models, but it is unclear which features are most relevant for accurate seizure detection and prediction.
– **Which ML and DL models perform best for epileptic seizure detection and prediction?** Several ML and DL models have been proposed for seizure detection and prediction, but it is unclear which models perform best and under what conditions.

By addressing these questions and hypotheses, we aim to provide a comprehensive understanding of the current state of the field of ML and DL methods for epileptic seizure detection and prediction and identify potential areas for future research.

## 2 Signal processing and feature extraction

EEG signals may be affected by various artifacts that can mask information related to epilepsy. The type and details of the EEG recording can influence the presence and nature of these artifacts.

A common step in processing EEG signals is removing background noise through filters to eliminate the 50–60 Hz powerline frequency. After this step, a large number of features can be extracted from the processed signal.

### 2.1 Methods

The present work is a narrative review [6], which is a traditional way of reviewing the existing literature on a particular topic. Narrative reviews attempt to summarize or synthesize what has been written on a particular topic but do not seek generalization or cumulative knowledge from what is reviewed. The primary goal of a narrative review is to determine the extent to which a body of literature provides evidence for a particular theory or hypothesis. Narrative reviews are helpful in obtaining a broad perspective on a topic. The present work aims to provide a comprehensive understanding of the current state of the field of epileptic seizure detection and prediction by summarizing and synthesizing the existing literature on the topic.

Internet bibliographical searches were conducted using the keywords “epileptic seizure prediction,” “artificial intelligence,” and “neural networks (computer)” to identify relevant literature on the topic. Through cross-referencing from key articles, additional references were obtained to provide a comprehensive overview of various artificial intelligence techniques for epileptic seizure prediction. The articles here referenced range from 1901 to 2022, and have been extracted from Web of Science and Scopus, along with the 142 databases available to the members of the Universidad de Salamanca [7].

This paper aims to present a detailed analysis of the current methodologies for the application of artificial intelligence techniques in epileptic seizure prediction, including classical ML algorithms, and DL algorithms such as dense neural networks (DNN), convolutional neural networks (CNNs), and recurrent neural networks (RNNs). As it is not intended to portray a pure bibliographical review of the subject, the publications here cited have been selected among many others based on their relevance and number of citations.

### 2.2 Feature extraction

#### 2.2.1 Statistical features

EEG signals can be analyzed using statistical parameters such as kurtosis, mean, skewness, and variance [8] to track changes in distribution and amplitude. Phase correlation [9, 10] can be used to examine patterns in ictal and preictal events, and Common Spatial Pattern [11] can extract features through the decomposition of EEG signals.

#### 2.2.2 Nonlinear features

Correlation Dimension [12, 13] can be used to measure the complexity of each event, and the Largest Lyapunov Exponent [14, 15] can calculate the level of chaos in EEG signals. Fractality Dimension [16, 17] can be used to compare rhythms between EEG events and identify self-similarity in the data. The Repeatability of the events can be quantified using Lempel-Zic Complexity [12, 18] and Approximate Entropy [19, 20]. Entropy can also be used as a measure of randomness with Spectral Entropy and level of disorganization using Wavelet Entropy.

The Hjorth parameters analysis [21, 22] includes activity (variance of the signal of a time function), mobility (proportion of the standard deviation), and complexity (change in frequency) as descriptors. Wavelet Energy [23, 24] can be used to examine the uniformity of different frequency bands in EEG data.

#### 2.2.3 Frequency domain features

Short Time Fourier Transform [25], and Fractional Fourier Transform [26] are used to calculate phase and magnitude components. Spectral Power Analysis [27, 28] can be used to study different frequency bands in EEG.

#### 2.2.4 Time-frequency domain features

The wavelet transform is a widely used time-frequency domain feature in the analysis of EEG. There are two types of wavelet transform: the Discrete Wavelet Transform [29] and the Continuous Wavelet Transform [30]. The CWT generates a scalogram through dilation and translation, while the DWT filters the signal and decomposes it into different levels.

Certain time-frequency domain features can be combined with nonlinear features to examine hidden data properties. Higher-Order Spectra [31, 32] and Variational Model Decomposition [33, 34] are among the most commonly used features in these combinations (Tables 1 and 2).

### 2.3 Feature selection

For most ML techniques to perform at their best, it is important to select an optimal number of features. Too many features that convey similar information about the target variable, or no information about it at all, can make the model unnecessarily complex and hinder its performance.

There are a variety of techniques that can be employed to identify the most suitable features for a particular study (Table 3). Statistical approaches such as Principal Components Analysis [36, 37] and Partial Least Squares [38] are relatively straightforward ways of reaching a conclusion. More complex techniques like Minimum Redundancy Maximum Relevance [39, 40] and Gaussian Mixture Models [41, 42] may be used in situations that require greater sensitivity, such as predicting human seizures.

However, some DL algorithms have the ability to automatically extract the most relevant information from the input variables using part of their architecture [43, 44]. Both feature extraction and selection can be bypassed in these cases and still yield competitive results.

## 3 Seizure detection and classification

Seizure detection using EEG has traditionally been performed manually by clinical professionals, who evaluate characteristics such as frequency, wavelength, voltage, amplitude, and waveforms. These features can be analyzed using automated learning algorithms. Automated detection of ictal events using EEG has become a crucial area of research in epilepsy since the first computer analysis of EEG records using wavelet transform in 2002 [47].

In recent years, numerous studies have been conducted to enhance the performance of automated seizure detection. A variety of ML and DL classifiers have been used [48]. Still, while detecting seizures may be helpful for research purposes, patients and clinical professionals require tools that can help prevent seizures rather than simply performing post-hoc analysis. This is where seizure prediction becomes necessary.

## 4 Seizure prediction

Having sufficient advance warning before a seizure occurs is a key aspect of clinical treatment. The process of seizure prediction is similar to seizure detection, but instead of classifying interictal and ictal events, it focuses on distinguishing interictal from preictal events, leaving out the actual seizures from the analysis.

Developing an appropriate stimulation protocol requires a sufficient advance warning before a seizure. However, early prediction often leads to reduced sensitivity and specificity, which can negatively impact overall performance (Table 4).

### 4.1 Animal models

The first experiments using wavelet functions as predictors achieved times of approximately 2.24 min and demonstrated their effectiveness [35]. The best time results in animal models were obtained by [45], which used canine EEG data and multiple ML algorithms to detect seizures 1 min in advance. While this work established a proof of concept, no diagnostic performance analyses were conducted. However, some studies have developed systems with sensitivities exceeding 90 %, although with somewhat longer prediction times. For example, Rajdev’s team (as described in [49]) developed a seizure prediction system for rat EEG recordings based on an adaptive Wiener filter, achieving a sensitivity of 92 %, which is the highest sensitivity achieved in works on animal EEG recordings.

### 4.2 Human subjects

The work of Iasemidis [50] established a maximum prediction time of 91 min with a precision of 91.3 % (and a sensitivity of 81.82 %). Other authors have sought to maintain a prediction time horizon within certain parameters. For example, in [12, 19], 30-min and 50-min horizons were fixed, resulting in sensitivities of 79.9 %–90.2 %.

Tsiouris [46] achieved a prediction with 15–120 min advance warning and a sensitivity of 99 %, using Long Short-term Memory networks (LSTM), which marked the first application of DL in this field.

Other DL approaches that automate feature extraction have achieved similar results. For example, Wei [43] used an image of the EEG as input to an architecture based on Convolutional Neural Networks (CNN) for feature extraction and Long Short-term Memory (LSTM) for sequence learning, achieving an average accuracy of 93.4 % with an average warning time of 21 min. Transformer models have also been used in a similar way [44], achieving a prediction sensitivity and False Positive Rate of 96.01 % and 0.047/h, respectively, with an average warning time ranging from 3 to 30 min.

## 5 Conclusions

Despite the significant progress made in automated seizure prediction since the first studies were conducted, there are still some challenges that need to be addressed.

To implement accurate diagnostic and treatment tools, it is necessary to achieve a balance between prediction time, sensitivity, and specificity. This balance can be achieved using DL techniques like CNNs, LSTMs, and Transformers. There have been some recent developments in this area that show promising results [43, 44, 51].

The best performance and results are often achieved by combining multiple techniques and features, but this approach can also increase computational requirements. Finding ways to effectively manage these requirements is a significant challenge in automated seizure prediction.

Finding a solution to the problem of automated seizure prediction has the potential to greatly improve the quality of life for epilepsy patients and reduce the burden on healthcare systems.
